# Effects of Polyurethane Foam Dressings as an Add-on Therapy in the Management of Digital Ulcers in Scleroderma Patients

**Published:** 2020-05-31

**Authors:** FW Rossi, F Rivellese, F Napolitano, F Mosella, C Selleri, N Montuori, A de Paulis

**Affiliations:** 1 Department of Translational Medical Sciences and Center for Basic and Clinical Immunology Research (CISI), University of Naples Federico II, Naples, Italy; 2 William Harvey Research Institute and Barts and The London School of Medicine and Dentistry, Queen Mary University of London, London, UK; 3 Department of General and Specialist Surgery, University of Naples Federico II, Naples, Italy; 4 Department of Medicine and Surgery, University of Salerno, Fisciano SA, Italy

**Keywords:** Systemic sclerosis, digital ulcers, Polyurethane

## Abstract

Digital ulcers (DUs) represent a severe and common complication occurring in patients affected by Systemic Sclerosis (SSc), with a consistent impact on the quality of life and often resulting in longer hospitalization than unaffected patients. Conventional treatment of SSc ulcers consists of both topical and systemic (oral or intravenous) pharmacological therapies. Several surgical options are also available, but there is overall a lack of official guidelines or recommendations.

The aim of this study was to evaluate the efficacy of a novel local therapy based on polyurethane foam dressings, namely the Highly Hydrophilic Polyurethane Foam (HPF), in addition to the conventional pharmacological treatment, in a cohort of 41 SSc patients with at least one active ulcer.

Our results showed that the addition of HPF to the conventional treatment based on systemic drugs induced i) a significant reduction in the number of active DUs (p=0.0034); ii) a significant reduction of the mean duration of ulcer-related hospitalization as compared with standard therapy (p=0.0001); iii) a significant improvement of patients’ Quality of Life, as evaluated through the Scleroderma Health Assessment Questionnaire (SHAQ) (p=0.00011).

Therefore, in our experience, the combined management of DUs can improve both the onset of new DUs and DU’s healing thus leading to a better outcome.

## I INTRODUCTION

Systemic sclerosis (SSc) is a chronic multisystemic disease characterized by immune deregulation with specific autoantibodies, vascular dysfunction and increased fibroblast activity resulting in diffuse fibrosis [1].

Digital ulcers (DUs) represent a very common and serious clinical manifestation of the vascular damage observed in SSc; they can indeed be considered as an end-organ damage resulting from progressive vasculopathy and a biomarker of disease activity and organ impairment [2]. At least one DU can occur approximately in half (44–60%) of affected patients [3].

These lesions severely limit patients’ everyday life, impacting over Quality of Life (QoL) and morbidity [4]. Ulcers can also have serious complications, such as infections and tissue loss, gangrene, osteomyelitis, potentially leading to amputation [4]. SSc patients with DUs often experience anxiety and a significant disability, representing a consistent economic burden due to a higher hospitalisation rate and the need of advanced therapies [5, 6].

DUs are commonly defined as areas of denuded tissue affecting the dermal and epidermal skin layers, usually involving fingertips; they can be multiple, recurrent, painful and difficult to heal [7,8]. Although authors use a variety of indicators to assess DU, such as size, number, location, loss of function, pain, infection and evolution to gangrene and time to healing, these items have been not yet fully validated. To define precisely what is an SSc ulcer is still an unmet need, despite DU prevention and healing have been primary endpoints in several clinical trials [9,10]. Recently Suliman *et al*., taking advantage from the World Scleroderma Foundation (WSF), developed a preliminary consensus based definition of SSc-skin ulcers [11].

In 2009 recommendations on management of digital vascular disease were published from EULAR experts [12]. The medical management of patients presenting this condition is often left to tertiary referral centers and is primarily conservative, involving both topic and systemic (oral or intravenous) therapies as first-line vasodilators (i.e. calcium channel blockers), Phosphodiesterase 5 inhibitors (PDE5i), Prostaglandin E1 (PGE1)/prostacyclin, Endothelin Receptor Antagonist (ERA), antiplatelet and antithrombin agents [13].

Many surgical options are also available, such as botulinum injections and digital sympathectomy [14]. However, clinicians are still lacking guidelines on the best management of DUs in SSc patients. In 2015, the UK Scleroderma Study Group developed three clinical algorithms intended to provide the best consensus recommendations for the management of SSc-specific complications, including digital vasculopathy [15].

The guidelines of the European Society for Vascular Medicine and an update of the EULAR recommendations for the management of Raynaud’s phenomenon (RP) have been recently published, however the evidence for their efficacy in the treatment of both primary and secondary RP is weak or moderate [16,17].

Highly Hydrophilic Polyurethane Foam (HPF) dressing is commonly used in clinical practice and its efficacy has been widely described in the treatment of diabetic foot ulcers [18] and pressure ulcers [19]. Polyurethane foam dressings have been used worldwide to accelerate wound healing, however no clinical studies demonstrate the efficacy of this novel foam dressing on scleroderma ulcers.

Aim of this study was to evaluate the effects of polyurethane foam dressings as an “add-on” therapy for SSc patients affected by DUs, on both number of ulcers and QoL.

## II. METHODS

The study is designed as a retrospective cohort study in which data are evaluated from the medical records of 41 SSc patients ≥18 year-old, admitted to the Department of Translational Medical Sciences - Autoimmune Diseases Unit of the University of Naples Federico II, classified according to the ACR/ EULAR criteria [20] and followed up during the period from January 2015 to January 2017. The main inclusion criterion was the presence of at least one active DU. Active DUs were determined by the physician and defined as a painful area of at least 2 mm in diameter characterized by visible depth and loss of dermis. DUs were localized in the palmar or dorsal surface of a finger, or sited next to an inter-phalangeal joint. No DU was associated with calcinosis. Patients with DUs not due to systemic sclerosis, and those affected by disorders that could represent confounding factors in assessing hand functionality (amputations, arthritis, other connective tissue diseases), were excluded from the study. Patients with diabetes, cancer, and heart or kidney failure were also excluded.

41 patients were consecutively enrolled and observed for 24 months. All patients underwent conventional therapy based on vasodilators (i.e. calcium channel blockers), PDE5i, PGE1/prostacyclin, ERA, antiplatelet and antithrombin agents (Table 1) for 12 months; polyurethane foam dressings treatment was then added for an additional 12 months. Since digital ulcers may be significantly painful, the use of Paracetamol as a painkiller, at the dose of one or two 500 mg tablets up to 4 times in 24 hours, was allowed. Medication with polyurethane foam dressing was changed daily after cleansing with a 0.9% saline solution. No other debridement of the ulcers was effectuated. All patients received a monthly routine check up.

The study was conducted in accordance with the principles of the Declaration of Helsinki and good clinical practice guidelines of the International Conference of Harmonization (ICH GCP); all patients gave their written informed consent and the study was approved by the ethical committee of the University of Naples Federico II (Protocol No. 348/2018, approved 14/03/18).

The overall physical disability was assessed by the Health Assessment Questionnaire for systemic sclerosis (SHAQ) [21].

Statistical analysis was performed by student T-test. Continuous variables are expressed as mean ± standard deviation; categorical variables are expressed as frequencies or percentages and differences were considered significant when *p* <0.05.

## III. RESULTS

**Fig. 1:**
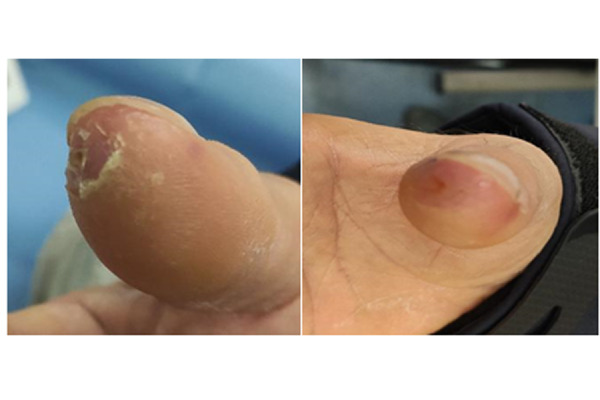
Digital ulcers in a male patient at T0 and after 4 weeks.

**Fig. 2:**
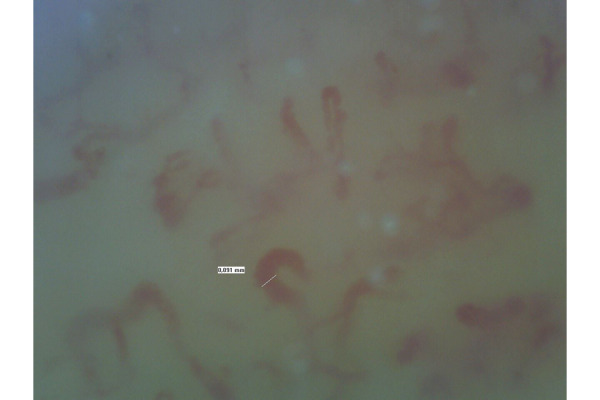
Nailfold exam at T0. It is possible to observe a late scleroderma pattern characterized by a severe capillary architecture disorganization with loss of capillaries, very few giant capillaries, absence of haemorrhages, and large avascular areas.

The characteristics of the study population are listed in Table 1: the majority of patients were female (85%) with the disease diagnosed more than a decade prior to the study (mean 15.6 yrs). The most common SSc subset was the diffuse one (54% vs 46%), with frequent positivity for Scl-70 topoisomerase I antibodies. Both groups experienced at least one active DUs (Fig. 1), and there was no significant difference between them concerning the clinical manifestations. Capillaroscopy was routinely performed: at T0 more than half the patients already showed a “late” pattern (Fig. 2). All patients underwent a combination therapy with Iloprost and vasodilators (100%), 37 patients (90%) were under antiplatelet agents and 68% of them also assumed ERA.

**Fig. 3:**
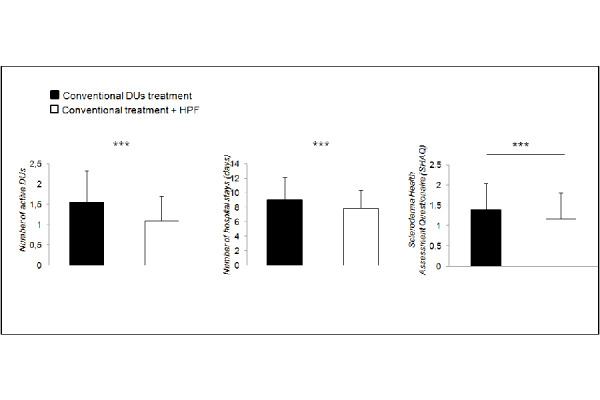
Number of active ulcers, hospitalization rates (expressed as days of hospital stay) and Scleroderma Heatlh Assessment Questionnaires (SHAQ), as indices of quality of life, are represented before and after the introduction of HPF as an add-on therapy. Each parameter results significantly reduced when patients undergo combined therapy. * p<0.05. List of abbreviations: DUs, Digital Ulcers; HPF, highly hydrophilic polyurethane foam. Data is expressed as mean +− standard deviation.

The HPF “add-on” treatment led to a significant reduction of the number of active DUs (mean 1,57 vs 1,09; p <0,0001), as shown in Figure 3A.

Also, the mean duration of ulcer-related hospitalization was significantly reduced following the addition of the polyurethane foam dressings (mean 9,07 days vs 7,87; p <0,0001, Figure 3B).

Moreover, while 2 patients under “traditional” therapy underwent amputations of phalanges before the introduction of the HPF treatment, no new amputations were registered in the year following the introduction of the HPF therapy.

Finally, Fig. 3 shows significantly improved scores (1,56 vs 1,09; p <0,0001) in SHAQ in the 12 months following the introduction of the HPF treatment (white columns) in comparison with the conventional therapies alone (black columns).

## IV. DISCUSSION

DUs are a very common visible expression of the progressive vascular damage that occurs in SSc usually requiring complex poly-therapy mainly based on systemic drugs and surgical approaches. Ulcers can also lead to amputation and debridement plays a crucial role to prevent further complications.

Debridement can be achieved through various methods (surgical, enzymatic, autolytic, mechanic, or biological) mostly depending on the extension of necrotic areas and on the patient’s compliance.

Although many official protocols share DUs pharmacological treatment [22], there is limited evidence to guide clinicians in the management of SSc-related digital vasculopathy. The UK Scleroderma Study Group produced recommendations for the management of SSc-specific complications, including digital vasculopathy [15].

Scrupulous consideration must be given to wound care of digital ulcers, especially in relation to the severity and the physical condition (e.g. wet or dry) of the ulcer. Moreover, while there is agreement about acute procedures [23], no indication is available on chronic maintenance.

Overall therapy is actually based on the everyday practice and can differ from one centre to another.

Polyurethane belongs to the group of hydro-active dressings. They are used in exuding wounds as they guarantee the removal of exceeding exudates, thus preventing the creation of a too dry environment. Polyurethane is a highly absorbent polymer that traps fluids, thus maintaining a damp setting. A good balance between moisturizing the wound site and absorbing exudate is essential for optimal wound healing because cell migration is encouraged, leading to re-epithelialization.

Like semi-permeable dressings, it protects perilesional skin from maceration thus preventing wound enlargement and pain increase. At the same time, it acts like a physical barrier, limiting bacterial colonization from the environment.

Polyurethane dressings are easy to handle especially when used on joints and fingers since they are ductile enough to expand and contract without blocking the wounded part. They also help to avoid micro-traumas during daily activities. Moreover, they are well tolerated by patients as they are “comfortable”.

In our experience, polyurethane foam dressings treatment proved successful in DUs healing in SSc patients. The close observation of exudates from wounds and the selection of appropriate dressing are crucial, considering the general cost and the wound management properties offered by each dressing type. We chose this kind of dressing first of all because it is not particularly expensive as compared to other similar devices currently used for the treatment of ulcers. Moreover, since SSc patients often have to deal with chronic ulcers, advanced polyurethane foam dressings can be easily handled at home, as this medication requires minimum management, thus consistently reducing the risk of infection and ensuing complications. It should also be taken into account that all SSc patients may undergo immunological therapy with immunosuppressive drugs because of multiorgan involvement. These drugs, lowering the body immune response, make it easier for infections to occur and to cause severe complication, such as amputation.

DUs, as one of the main complications, represent a consistent burden not only to SSc patient but also to society: besides affecting patient’s working ability to cope with common daily activities, they also have a consistent economic cost since DUs treatment requires frequent hospitalization and highly expensive therapies [24].

As a matter of fact, the introduction of polyurethane foam dressings considerably reduced the average length of hospitalization as observed over one-year follow-up. A reduced need for hospital admissions emerged as well, since the simple polyurethane foam dressing application can actually be performed at home, allowing patients to be dismissed earlier, consequently reducing both direct and indirect DU-related healthcare costs.

As reported, a change in digital ulcers status was accompanied by an improvement in the QoL patient reported outcome [21]; indeed, we observed a significant change in the SHAQ score with a better global health status.

Scleroderma-related DUs are a serious cause of morbidity. The search for well-tolerated, inexpensive therapeutic options for the treatment of this complication is still an unmet need.

In order to assess and confirm the efficacy of HFP as an add-on treatment in patients affected by DUs in the context of SSc, a larger sample of patients may be required. What emerges from this study is that the combined management of DUs, as compared to pharmacological therapy alone, can favour their healing. Moreover, the combined therapy determines not only a decrease in the number of DUs complications, but also a reduced hospitalization rate and an improvement of the quality of life, thus leading to a better outcome.

## Figures and Tables

**Table 1:** Demographics and clinical parameters of the study population.

*Clinical features of SSc patients*	

Women	35 (85%)
Men	6 (15%)
Age, mean ±SD years	51,9 (± 14.5)
Disease duration, mean ±SD years	15,6 (± 11.5)
Local SSc (lSSc)	19 (46%)
Diffuse SSC (dcSSc)	22 (54%)
Puffy fingers	31 (76%)
DLCO <80%	12 (28%)
Dyspnoea	14 (34%)
ACA positive	20 (49%)
Anti-Scl 70 positive	21 (51%)
NVC early pattern	8 (19%)
NVC active pattern	10 (24%)
NVC late pattern	23 (57%)
CCB	39 (95%)
PDE5i	3 (7%)
PGE1/prostacyclin	38 (93%)
ERA	28 (68%)
Antiplatelet agents	37 (90%)

Data are expressed as percentages.

List of abbreviations: SSc, Systemic Sclerosis; ACA, anti-centromere antibodies; anti-Scl70 antibodies, anti-DNA Topoisomerase I, NVC, Nailfold video-capillaroscopy; CCB, Calcium channel blockers; PDE5i, Phosphodiesterase 5 inhibitors; PGE1, prostaglandin E; ERA, endothelin receptor antagonist.
